# Addition of Partial Envelope Domain II into Envelope Domain III of Dengue Virus Antigen Potentiates the Induction of Virus-Neutralizing Antibodies and Induces Protective Immunity

**DOI:** 10.3390/vaccines8010088

**Published:** 2020-02-15

**Authors:** Jisang Park, Hyun-Young Lee, Ly Tuan Khai, Nguyen Thi Thu Thuy, Le Quynh Mai, Yong-Suk Jang

**Affiliations:** 1 Department of Bioactive Material Sciences and Research Center of Bioactive Materials, Jeonbuk National University, Jeonju 54896, Korea; 2 Department of Hematology, 108 Military Central Hospital, Hanoi 113601, Vietnam; 3 National Institute of Hygiene and Epidemiology, Hanoi 100000, Vietnam; 4 Department of Molecular Biology and The Institute for Molecular Biology and Genetics, Jeonbuk National University, Jeonju 54896, Korea

**Keywords:** dengue virus, subunit vaccine, antibody-dependent enhancement, antibody

## Abstract

Dengue virus (DENV) comprises four serotypes in the family *Flaviviridae* and is a causative agent of dengue-related diseases, including dengue fever. Dengue fever is generally a self-limited febrile illness. However, secondary infection of patients with a suboptimal antibody (Ab) response provokes life-threatening severe dengue hemorrhagic fever or dengue shock syndrome. To develop a potent candidate subunit vaccine against DENV infection, we developed the EDII-cEDIII antigen, which contains partial envelope domain II (EDII) including the fusion loop and BC loop epitopes together with consensus envelope domain III (cEDIII) of all four serotypes of DENV. We purified Ab from mice after immunization with EDII-cEDIII or cEDIII and compared their virus neutralization and Ab-dependent enhancement of DENV infection. Anti-EDII-cEDIII Ab showed stronger neutralizing activity and lower Ab-dependent peak enhancement of DENV infection compared with anti-cEDIII Ab. Following injection of Ab-treated DENV into AG129 mice, anti-EDII-cEDIII Ab ameliorated DENV infection in tissues with primary and secondary infection more effectively than anti-cEDIII Ab. In addition, anti-EDII-cEDIII Ab protected against DENV1, 2, and 4 challenge. We conclude that EDII-cEDIII induces neutralizing and protective Abs, and thus, shows promise as a candidate subunit vaccine for DENV infection.

## 1. Introduction

Dengue virus (DENV) is an arthropod-borne single positive-strand RNA virus and a causative agent of several diseases [1,2]. There are four antigenically-related serotypes of DENV. Since the 1950s, geographical expansion of DENV-infected diseases has occurred due to the global warming-induced increase in the area suitable as habitat for its host mosquitoes (*Aedes aegypti* and *Aedes albopictus*). Over 390 million people are estimated to be infected with DENV annually, 96 million of whom show clinical symptoms [1,2,3]. DENV causes a spectrum of conditions from self-limited mild dengue fever to severe dengue hemorrhagic fever and dengue shock syndrome [1,2]. Because the immune response induced following infection with a specific serotype of DENV has poor cross-reactivity with other serotypes, people who recovered from infection with one serotype of DENV are not protected against infection with other serotypes [4,5]. Those secondary heterotypic infections often induce more severe clinical disease than primary infections due to antibody (Ab)-dependent enhancement (ADE) of infection, where virus-bound Ab promotes the entry of the virus into myeloid cells [5]. ADE hampers DENV vaccine development; hence, a DENV vaccine must induce neutralizing Abs to the four serotypes of DENV but not non-neutralizing Abs to prevent ADE.

Much effort has focused on developing vaccines effective against the four serotypes of DENV. Dengvaxia is produced by Sanofi Pasteur, although there are reports of complications [6]. Dengvaxia contains four chimeric live flaviviruses, each derived from the genome of the yellow fever virus 17D vaccine strain; however, the precursor membrane (prM) and envelope (E) gene segments are replaced by those of the four DENV serotypes [6,7]. Dengvaxia is effective against the four serotypes of DENV, albeit weakly against serotype 2 (DENV2), but increased hospitalization was reported in children under 9 years of age that were vaccinated with Dengvaxia compared to unvaccinated children after subsequent infection with DENV, possibly due to ADE, a “Dengvaxia controversy” [8,9,10]. The United States National Institutes of Health developed a live attenuated tetravalent vaccine containing the recombinant genomes of four DENV strains. The DENV vaccine strains were attenuated by deleting 30 nucleotides from the 3′-untranslated region, and the DENV2 strain was a chimera of the DENV4 genome with the prM and E genes of DENV2 [11]. Additionally, DENVax (Takeda) comprises four recombinant DENV2-based viruses in which the prM and E gene segments are replaced by those of DENV1, DENV3, and DENV4 [12]. These candidate vaccines have shown promise in clinical trials with good results. Given that virus-based vaccines may trigger concerns about the potential reversion of pathogenicity, alternative approaches are needed to develop a DENV vaccine [13].

One such alternative is a recombinant protein antigen (Ag)-based subunit vaccine. Subunit vaccines are typically safer than attenuated virus vaccines and are capable of inducing an immune response against the four DENV serotypes [14,15,16]. A subunit vaccine with restricted epitopes can be used to reduce unwanted Ab production, which is not involved in virus neutralization, and so prevents ADE [17,18]. Additionally, a subunit DENV vaccine could provide complete protection more rapidly than a live attenuated DENV vaccine [16]. The DENV E protein and its envelope domain III (EDIII) have been investigated as candidate Ags for recombinant subunit vaccine using various approaches (e.g., fusion with other components of viral Ags and use of adjuvants) [19]. The leading subunit vaccine candidate is V180, which was developed by Merck and consists of recombinant carboxyl-truncated E proteins (DEN-80E) of the four DENV serotypes. It induces a high titer of neutralizing Abs and provides protection against the four DENV serotypes in mice and rhesus monkeys [14]. Additionally, because EDIII contains the receptor-binding site, its suitability as a vaccine antigen has been investigated [20]. The fusion loop epitope (FLE) and BC loop epitope of envelope domain II (EDII) were recently found to have a conserved sequence and induce neutralizing Abs capable of protecting against the four DENV serotypes [21,22,23]. Therefore, both EDII and EDIII are important to induce neutralizing Abs against the four DENV serotypes and show promise for a DENV vaccine.

Consensus EDIII (cEDIII) is a potential candidate Ag for a DENV vaccine. The induced Abs protected against the four DENV serotypes, although the neutralizing capacities against DENV3 and 4 were lower than those against DENV1 and 2 [24]. In this study, we conjugated EDII (50–130 aa) of DENV4 to the N-terminus of cEDIII to improve the protective ability of the Abs induced by the Ag because EDII (50–130) contains two major (FLE and BC loop) epitopes related to neutralization of the virus. We assessed the efficacy of EDII-cEDIII as a candidate vaccine Ag by comparing the neutralizing activity and ADE induction of the Ab induced by EDII-cEDIII with that induced by cEDIII in vitro using Vero E6 and dendritic cell-specific intercellular adhesion molecule-3-grabbing non-integrin-expressing U937 (U937-DC-SIGN) cells. Additionally, the neutralizing activity of the Abs was compared by injecting Ab-treated DENV into AG129 mice, an animal model of DENV infection. Finally, the ability of EDII-cEDIII to protect against DENV challenge was evaluated by determining the DENV titer in blood and tissues.

## 2. Materials and Methods

### 2.1. Experimental Materials and Animals

Unless otherwise specified, the chemicals and laboratory wares used were purchased from Sigma Chemical Co. (St. Louis, MO, USA) and SPL Life Sciences (Pocheon, Korea), respectively. Oligonucleotide primers were purchased from Macrogen Inc. (Seoul, Korea). AG129 mice, which are deficient in type I and II interferon (IFN) receptors, were purchased from Marshall BioResources (Hull, East Yorkshire, UK) and maintained under specific pathogen-free conditions with ad libitum access to food and water. A group of three to five mice was used in each experiment, and the procedures involving animals were approved by the Institutional Animal Care and Use Committee of Chonbuk National University (Approval Number: CBNU 2017-0075) and followed the guidelines suggested by the committee.

### 2.2. Cell Culture

Vero E6 cells were purchased from the American Type Culture Collection (ATCC; Manassas, VA, USA) and were used for viral neutralization assays and to propagate DENV. Vero E6 cells were cultured in Dulbecco’s modified Eagle’s medium (DMEM; Welgene, Gyeongsan, Korea) supplemented with 5% fetal bovine serum (FBS; Gibco, Thermo Fisher Scientific, Grand Island, NY, USA). In addition, U937-DC-SIGN cells (ATCC, U937 cells transfected with the human DC-SIGN gene, encoding an attachment factor for DENV) were used for the DENV binding and ADE experiments. U937-DC-SIGN cells were cultured in Roswell Park Memorial Institute (RPMI) medium (Welgene) containing 5% FBS, 2 mM L-glutamine, 0.1 mM non-essential amino acids, and 0.05 mM 2-mercaptoethanol.

### 2.3. Production of Recombinant Ags

DNA encoding the 103 amino acid (aa) cEDIII protein was synthesized as described previously [25]. To amplify the partial EDII (50–130 aa, 1062–1304 nt) gene and partial EDII hinge region (264–279 aa, 1710–1757 nt) (GenBank: MF004387.1), cDNA was prepared from DENV4-infected cells. We used polymerase chain reaction (PCR) to amplify the partial EDII (50–130) gene from DENV4 cDNA using a forward primer (5′-GAG CTC GCC AAG GAA GTG GCT CTG TTA-3′; underlined letters indicate the *Sac*I restriction site) and reverse primer (5′-TGC GTA AGA CAT TCC TTT CAT GAC CAA ATT GCC TGT TAT CTT-3′) containing the partial cEDIII sequence. The forward primer used to amplify cEDIII and containing the partial EDII sequence was 5′-AAG ATA ACA GGC AAT TTG GTC ATG AAA GGA ATG TCT TAC GCA-3′. The reverse primer used to amplify cEDIII was 5′-GGA TCC TTA *TCC TGC AAA CAT GTG ATT TCC ATC ACC GGA GTC CAC TTC TGT GGC TCC* TGA GGA ACC CTT TTT AAA CCA-3′ (underlined and italicized letters indicate the *Bam*HI restriction site and the EDII hinge region sequence of DENV4 (264–279), respectively). The two amplified fragments were combined to generate the EDII-cEDIII recombinant gene, which was then cloned into the pCold II *E. coli* expression vector (TaKaRa Bio, Shiga, Japan). The recombinant cEDIII and EDII-cEDIII genes were expressed in C43 competent cells (Lucigen Co., Middleton, WI, USA) and purified using Ni-NTA agarose (Qiagen, Hilden, Germany) as described previously [26]. The identity of the recombinant Ags was confirmed by sodium dodecyl sulfate–polyacrylamide gel electrophoresis and Western blotting using anti-DENV (EMD Millipore, Burlington, MA, USA) and 6×His tag (Qiagen) Abs.

### 2.4. Immunization and Ab Purification

Five-week-old AG129 mice underwent three consecutive immunizations in 2-week intervals via intraperitoneal injection of 100 μg Ag in 100 μL PBS emulsified in 100 μL alum (total 200 μL) (Thermo Fisher Scientific, Waltham, MA, USA). Three and 7 days after the final immunization, sera were collected and combined, and Abs were purified using Protein G-Magnetic Beads (GenScript, Piscataway, NJ, USA). Ag-specific Ab concentrations were determined by enzyme-linked immunosorbent assay (ELISA) as described previously [26], using EDII-cEDIII Ag-coated wells of Maxisorp Immunoplates (Nunc, Thermo-Fisher Scientific, Roskilde, Denmark).

### 2.5. Virus Propagation, Titration, and Neutralization

To propagate DENV, Vero E6 cells were infected with a small volume of DENV at a multiplicity of infection (MOI) of 0.1 in 2% FBS-containing medium. After incubation for 2 h at 37 °C, culture medium containing 2% FBS and HEPES (to prevent acidification of the medium and low pH-induced inactivation of newly released virions) was added. The incubation was continued at 37 °C for 4 days and the culture medium was harvested. The virus particles were concentrated by centrifugation at 30,000 × *g* for 2 h at 4°C, and the concentrated virus was stored as aliquots at −80 °C [27].

To determine the virus titer, 1.5 × 10^4^ Vero E6 cells were plated into the wells of a 96-well plate 1 day before DENV infection. The cells were infected with serial dilutions of the samples and incubated for 1 h at 37 °C in a 5% CO_2_ incubator and then incubated in overlay medium (Opti-MEM [Gibco] with 2% FBS, antibiotics, and 1% methylcellulose) for 3 days at 37 °C in a 5% CO_2_ incubator. The cells were fixed in methanol and acetone at room temperature for 30 min, blocked with 2% bovine serum albumin (BSA), and incubated with 100 μL primary anti-DENV Ab (EMD Millipore) for 2 h at room temperature. Finally, horseradish peroxidase-conjugated anti-mouse IgG Ab (Cell Signaling, Danvers, MA, USA) was added, and the color was developed using TruBlue Peroxidase Substrate (Seracare, Milford, MA, USA). The number of developed spots was counted to determine the number of focus-forming units (FFUs) of DENV.

To perform focus reduction neutralization tests (FRNTs), purified Abs were mixed with concentrated DENV and incubated for 1 h at 37 °C. Next, the mixture was applied to Vero E6 cells (to form approximately 100–150 spots per well), which had been plated in a 96-well plate 1 day prior, and incubated for 1 h at 37 °C. After washing with PBS, overlay medium was added, and the plate was incubated for 3 days. Finally, the spots were developed to count FFUs, and FRNT was calculated relative to that of the untreated samples and expressed as a percentage.

### 2.6. Ab Binding Assay

To assay DENV-binding Ab, the wells of a 96-well plate were coated with concentrated DENV and inactivated by UV irradiation for 30 min, followed by washing with 0.05% Tween-20 in PBS. After blocking with 2% BSA in PBS for 2 h at room temperature, purified Abs were titrated with the virus. Bound Abs were detected using alkaline phosphatase-conjugated anti-mouse IgG Ab and phosphatase substrate. The absorbance at 405 nm was measured using an ELISA reader (BMG Labtech, Ortenberg, Germany).

### 2.7. Neutralization Assay Using U937-DC-SIGN Cells

To perform neutralization assays using Ab against DENV in U937-DC-SIGN cells, DiD-labeled DENV was used. To label the virus with DiD, approximately 10^8^ FFU DENV were concentrated to 50 μL, to which 5 μL 1 mM DiD solution in DMSO was added, and the mixture was incubated for 20 min at room temperature. Unbound dye was removed using a Sephadex G-50 column (GE Healthcare, Chicago, IL, USA) prepared in short low-binding plastic pipette tips (Sorenson Bioscience, Salt Lake City, UT, USA). The DiD-labeled virus was collected, stored at 4 °C, and used within 2 days [28]. DiD-labeled DENV was mixed with Abs for 1 h at 37 °C, and the mixture was added to U937-DC-SIGN cells for 1 h at 37 °C. After washing with PBS, the cells were assayed using the CytoFLEX flow cytometer (Beckman-Coulter, Brea, CA, USA) and analyzed using FlowJo software (Tree Star, Ashland, OR, USA).

### 2.8. DENV Challenge Infection in AG129 Mice

Purified Ab (10 μg in 100 μL PBS) was intraperitoneally injected into age- and weight-matched AG129 mice before viral infection. The next day, purified Ab (10 μg in 100 μL PBS) was incubated with DENV1 (10^8^ FFU), DENV2 (10^7^ FFU), DENV3 (5 × 10^7^ FFU), or DENV4 (5 × 10^8^ FFU) for 1 h at 37 °C, and the mixture (Ab in 100 μL PBS and DENVs in 100 μL PBS) was then intraperitoneally injected into AG129 mice. We monitored the mortality of the mice. Axillary lymph nodes (LNs) and spleen were isolated 1 day after the DENV challenge to confirm the initial level of DENV infection. Blood was collected 3–4 days after the DENV challenge to prepare serum and blood cells, and liver and intestine samples were isolated and stored at −80 °C.

### 2.9. Blood Cell Isolation and Serum Cytokine Analysis

Blood was collected from DENV1–4-challenged mice and treated with ACK lysis buffer (Gibco) for 30 min at room temperature to remove red blood cells. Cells were resuspended in 2% paraformaldehyde for fixation and blocked with BSA and a FcγR blocking Ab. Cells were stained with phycoerythrin (PE)-conjugated anti-CD3, PE-conjugated anti-CD19, allophycocyanin (APC)-conjugated anti-Ly6C, PE-Vio770-conjugated anti-Ly6C, APC-Vio770-conjugated anti-CD11b, and peridinin–chlorophyll-protein (PerCP)-conjugated anti-CD11c (Miltenyi Biotec, Bergisch Gladbach, Germany) Abs. To detect intracellular DENV, marker-stained cells were permeabilized using Perm buffer (BD Biosciences, San Jose, CA, USA) and stained with a fluorescein isothiocyanate (FITC)-conjugated anti-DENV Ab (EMD Millipore). FITC conjugation was achieved using the IgG2a-FITC Conjugation Kit (Invitrogen, Thermo Fisher Scientific, Grand Island, NY, USA). Stained cells were assayed using the CytoFLEX flow cytometer (Beckman-Coulter), and the data were analyzed using FlowJo software (Tree Star). The gate for DENV Ag-positive cells was <1% compared with isotype control staining.

### 2.10. Reverse-Transcription Quantitative PCR (RT-qPCR)

The numbers of DENV gene copies and the cytokine levels were determined using RT-qPCR. Tissues and cells were harvested, and RNA was extracted using the easy-BLUE Total RNA Extraction Kit (Intron Biotechnology, Sungnam, Korea). RNA was converted into cDNA using a reverse-transcription reaction system (Promega, Fitchburg, WI, USA). RT-qPCR was performed in a 96-well plate with 2 pmol forward and reverse primers using SsoAdvanced Universal SYBR Green Supermix (Bio-Rad Laboratories, Hercules, CA, USA) on the CFX Connect Real-Time PCR Detection System (Bio-Rad) under conditions described previously [29]. Gene expression levels were normalized to the 18S rRNA level. The following forward and reverse primer sets were used: m18S RNA, 5′-TGC GCC GCT AGA GGT GAA ATT CTT-3′ and 5′-CAA ATG CTT TCG CTC TGG TCC GT-3′; mIFN-γ, 5′-ACT ACC TTC TTC AGC AAC AGC-3′ and 5′-TTG TTG ACC TCA AAC TTG GCA-3′; DENV1, 5′-GGA ACA TCC ATC ACC CAG AAA-3′ and 5′-ACG AAG TCT CTG TTG CCT ATT C-3′; DENV2, 5′-TGT GCA ACA CCG CAT AGT-3′ and 5′-GTC CCT GAC CTT GAA CCT AAT C-3′; DENV3, 5′-ACA GGC AAC ATC GTC TCT TC-3′ and 5′-CCT GCT CCT AAA TCC ACA TCT T-3′; and DENV4, 5′-GTT CGA CTG GAT AAC CGA CTA C-3′ and 5′-CAC CTT CTT TCC CGA CTT TCT-3′.

### 2.11. ADE Assay Using U937 Cells

DiD-labeled DENV was mixed with or without sample Ab for 1 h at 37 °C, and the mixture was applied to U937 cells for 1 h at 37 °C. After washing the cells with PBS, the DiD-labeled DENV-positive cell population was analyzed by flow cytometry. Fold enhancement was determined by comparing the value obtained with Ab treatment to that without Ab treatment. Peak enhancement was the highest fold enhancement induced by Ab treatment among the various Ab concentrations tested.

### 2.12. Statistical Analysis

Statistical analysis was performed using Prism 5 (GraphPad Software, La Jolla, CA, USA). Data are the means ± standard error (SE) of the values obtained from repeat experiments unless otherwise specified. Differences in the means of multiple independent variables were compared between the control and treatment groups using unpaired two-tailed *t*-tests. Differences in mean values were considered significant at *p* < 0.05.

## 3. Results

### 3.1. EDII of DENV4 Enhanced the Induction of Abs Capable of Binding to DENV4 and Neutralizing DENV3 and DENV4

**Figure 1:**
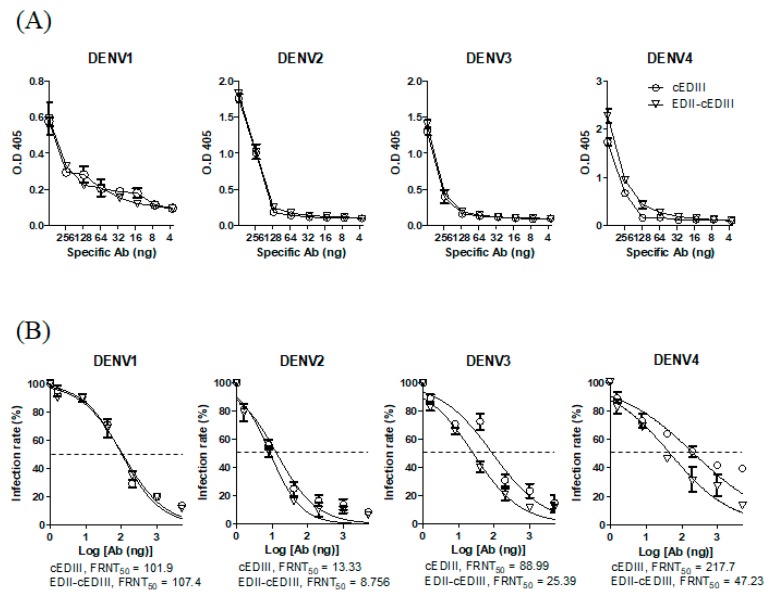
Effects of antibodies (Abs) on dengue virus (DENV) infection in Vero E6 cells. (**A**) Binding of Ab to DENV was measured using purified IgG against consensus envelope domain III (cEDIII) and envelope domain II (EDII)-cEDIII by enzyme-linked immunosorbent assay (ELISA) of the endpoint titer. Circles and inverted triangles represent anti-cEDIII and anti-EDII-cEDIII Ab-treated groups, respectively. (**B**) Neutralization endpoint titer of purified Ab against DENV serotypes by foci reduction neutralization assay in Vero E6 cells. Nonlinear regression (curve fit) was performed using GraphPad Prism software. Dotted line, 50% infection rate (FRNT_50_) calculated using Prism software. A representative result from three to five independent experiments is shown.

We first analyzed the binding of the purified Abs to each serotype of DENV (Figure 1A). Ab induced by EDII-cEDIII showed comparable binding to cEDIII, although anti-EDII-cEDIII Ab showed approximately 1.5-fold greater binding to DENV4 than anti-cEDIII Ab. Next, we compared the virus-neutralizing activity of the Abs in Vero E6 cells by performing FRNT to determine the FRNT_50_ values (Figure 1B). In accordance with the binding result, the neutralizing activity of anti-EDII-cEDIII Ab against DENV4 was about fourfold higher than that of anti-cEDIII Ab. Interestingly, although there was no difference in binding between the anti-EDII-cEDIII and anti-cEDIII Abs, the neutralizing activity of anti-EDII-cEDIII Ab against DENV3 was about threefold higher than that of anti-cEDIII Ab. However, there was no difference in the neutralizing activity of the Abs against DENV1 and DENV2. Therefore, addition of EDII of DENV4 enhanced the induction of Abs capable of binding to DENV4 and neutralizing DENV3 and DENV4.

### 3.2. Anti-EDII-cEDIII Abs Neutralized the Four DENV Serotypes in U937-DC-SIGN Cells

**Figure 2:**
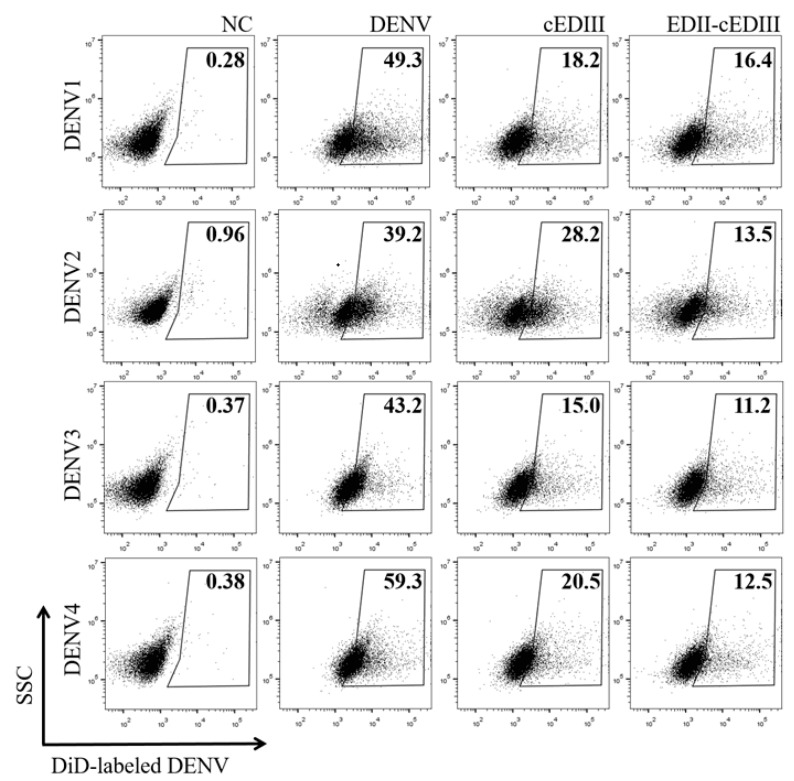
Neutralization of DENV infection in U937-DC-SIGN cells by Ab in vitro. Neutralizing activity was assessed by evaluating the effects on DENV infection in U937-DC-SIGN cells. *X*- and *y*-axes represent side scatter (SSC) and the level of DiD-labeled DENV, respectively. Percentage of DENV-positive cells is shown in the gating box. NC (negative control; no infection), DENV (only DENV infection), cEDIII (DENV and anti-cEDIII Ab), EDII-cEDIII (DENV and anti-EDII-cEDIII Ab). A representative result of three independent experiments is shown.

DENV uses a variety of receptors to bind to host cells, and the binding ligands in E protein vary depending on the receptors used [30]. Vero E6 cells have heparan sulfate as the receptor for virus infection, to which EDIII binds [30,31]. In contrast, N67 and N153 of E protein are important for DENV binding to mannose receptor or DC-SIGN [30,32]. Consequently, the virus neutralization activity of Abs induced by EDII-containing Ags was determined using DC-SIGN-expressing cells (Figure 2). Around 40–60% of U937-DC-SIGN cells became infected with various serotypes of DENV. Additionally, anti-EDII-cEDIII Ab reduced infection by DENV1, 2, 3, and 4 in U937-DC-SIGN cells by 66.8, 65.6, 74.1, and 79%, respectively, whereas anti-cEDIII Ab reduced infection by 62.4, 28.1, 65.3, and 65.5%, respectively. Although the neutralizing activity of the two Abs was similar in Vero E6 cells, the neutralizing activity of anti-EDII-cEDIII Ab was more potent than that of anti-EDIII Ab in DC-SIGN-expressing cells.

### 3.3. ADE Was Triggered by Anti-EDII-cEDIII and Anti-cEDIII Abs

**Figure 3:**
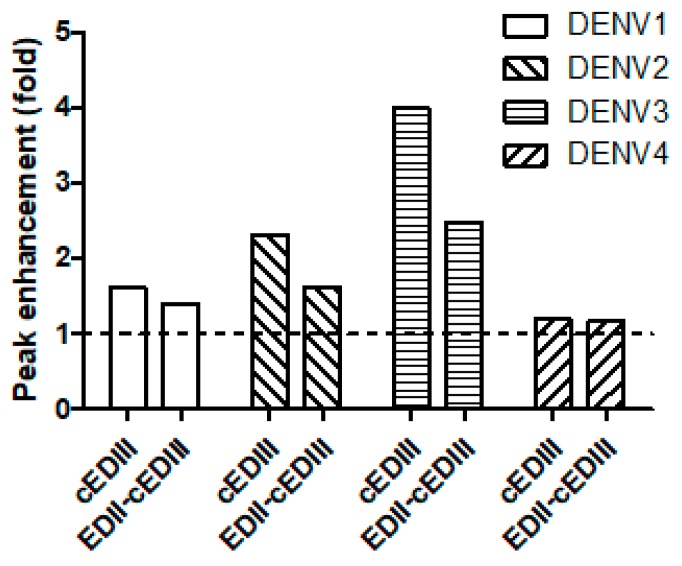
Antibody-dependent enhancement (ADE) of DENV infection by Ab treatment in U937 cells. Fold enhancement was determined by comparing the value obtained by Ab treatment with that obtained without Ab treatment. Peak enhancement was the highest fold enhancement induced by Ab treatment among the various Ab concentrations tested. The dotted line represents the fold change relative to DENV infection without Ab treatment. A representative result from two independent experiments is shown.

Abs against DENV induce ADE in vitro at low concentrations [33]. Because the degree of ADE varies depending on the Abs used [34], we compared ADE mediated by the Ab induced by EDII-cEDIII versus the Ab induced by cEDIII (Figure 3). Although the degree of ADE varied depending on the DENV serotypes tested, we observed ADE in all serotypes of DENV (Supplementary Figure S1). Interestingly the peak enhancement, which represents the strongest enhancement, was lower with the Ab induced by EDII-cEDIII than by cEDIII in various serotypes of DENV, with the exception of DENV4 (Figure 3). In particular, ADE was induced most strongly in DENV3, and a 1.5-fold lower peak enhancement was observed with the Ab induced by EDII-cEDIII than by cEDIII. Therefore, we suggest that the Ab induced by EDII-cEDIII induced lower ADE than did the Ab induced by cEDIII.

### 3.4. Anti-EDII-cEDIII Ab Increased the Survival of DENV-Infected Mice

**Figure 4:**
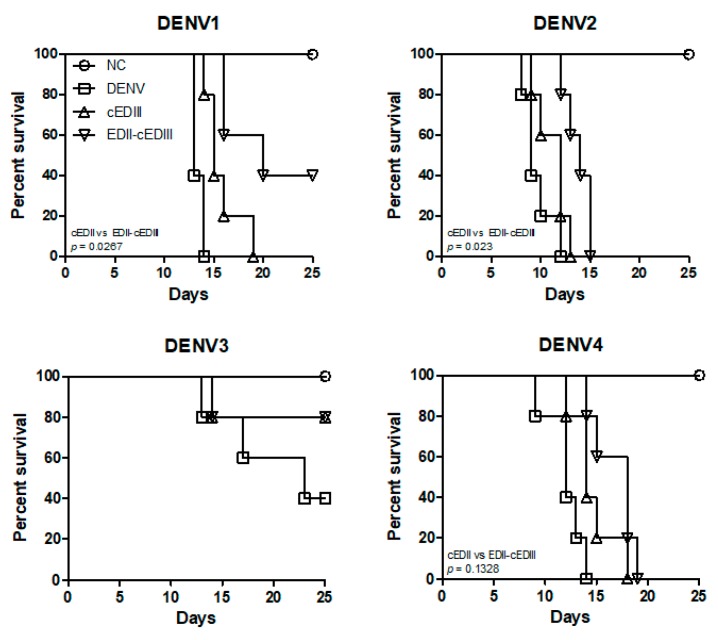
Purified Ab increases the survival of DENV-infected AG129 mice. Survival of AG129 mice after intraperitoneal administration of DENV in the presence of purified Ab or PBS was determined. Abs were intraperitoneally injected on day −1 and day 0 together with DENV. *p*-values between two groups were calculated by log-rank test, n = 5 mice per group. Squares, DENV; triangles, anti-cEDIII Ab treatment group; inverted triangles, anti-EDII-cEDIII Ab treatment group. A representative result from three independent experiments is shown.

Given that anti-EDII-cEDIII Ab was more effective than anti-cEDIII Ab against DENV infection in vitro, we conducted challenge infection in AG129 mice to evaluate the effects of anti-EDII-cEDIII and anti-cEDIII Abs on survival (Figure 4). Interestingly, anti-EDII-cEDIII Ab enhanced the survival of mice after challenge with various DENV serotypes. The survival of the AG129 mice was prolonged by the anti-EDII-cEDIII and anti-cEDIII Abs compared to control mice. The survival of DENV1- and DENV2-challenged mice was significantly (*p* < 0.0267 and 0.023, respectively) prolonged by the anti-EDII-cEDIII Ab compared to the anti-cEDIII Ab. Additionally, 40% of AG129 mice treated with anti-EDII-cEDIII Ab and challenged with DENV1 survived, compared to 0% of those treated with anti-cEDIII Ab. Although challenge with DENV3 did not induce strong lethality, the anti-EDII-cEDIII and anti-cEDIII Abs prolonged survival of the mice after challenge. Therefore, the protective efficacy of anti-EDII-cEDIII Ab against DENV challenge was superior to that of anti-cEDIII Ab. 

### 3.5. Anti-EDII-cEDIII Ab Reduced Initial DENV Levels in Axillary Lymph Nodes and Spleen

**Figure 5:**
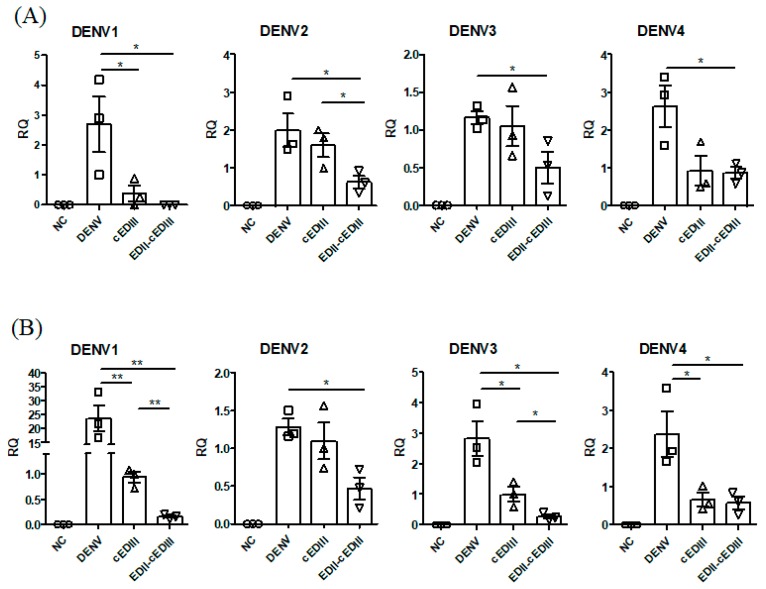
Purified Ab treatment reduces DENV infection at initial infection sites, axillary lymph nodes (LN), and spleen. Viral RNA levels in (**A**) axillary LN and (**B**) spleen were quantified by RT-qPCR 18–24 h after DENV infection of AG129 mice in the presence or absence of the indicated Ab. Data are means ± SE, and a representative result from two independent experiments is shown. Unpaired two‑tailed *t-*test. * *p* < 0.05 and ** *p* < 0.01 indicate significant differences. Squares, triangles, and inverted triangles represent the results from DENV-, anti-cEDIII Ab, and anti-EDII-cEDIII Ab-treated groups, respectively.

Anti-EDII-cEDIII Ab prolonged survival only in mice challenged with DENV1. We assumed this was due to limited persistence of the Abs and lack of production of effective Abs. Nevertheless, the prolonged survival caused by anti-EDII-cEDIII Ab suggested inhibition of initial DENV infection. To evaluate this, we assayed the DENV titer in axillary LNs (Figure 5A) and spleen (Figure 5B) by RT-qPCR 1 day after DENV infection, because DENV infection was first detected in forelimb LNs and spleen after intraperitoneal infection of AG129 mice [34]. Initial infection by the four serotypes of DENV in axillary LNs was significantly inhibited by anti-EDII-cEDIII Ab compared to the control (Figure 5A). Interestingly, DENV1 was not detected in the axillary LNs of mice treated with the anti-EDII-cEDIII Ab. In addition, initial infection of DENV2 of axillary LNs was inhibited by both Abs, although that caused by the anti-EDII-cEDIII Ab was significantly greater than that by the anti-cEDIII Ab. Although there was no significant difference in DENV3 and DENV4 infection in axillary LNs between the two Abs, only the anti-EDII-cEDIII Ab significantly reduced initial infection by DENV3 and DENV4 compared to the control. Similar, but greater, inhibition of initial DENV infection was also observed in the spleen (Figure 5B). Initial infection of the spleen by the four serotypes of DENV was significantly inhibited by the anti-EDII-cEDIII Ab. Moreover, inhibition of DENV1 and DENV3 infection of the spleen by the anti-EDII-cEDIII Ab was significantly greater than that by the anti-cEDIII Ab. The anti-EDII-cEDIII Ab inhibited DENV2 infection of the spleen more effectively than the anti-cEDIII Ab. Therefore, the anti-EDII-cEDIII Ab inhibited initial DENV infection in AG129 mice.

### 3.6. Anti-EDII-cEDIII Ab Inhibited DENV Infection of Lymphocytes

**Figure 6:**
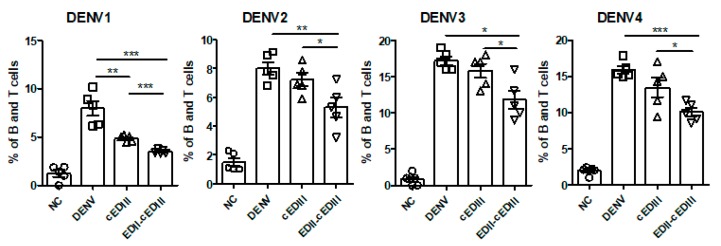
DENV infection of blood cells is inhibited by purified Abs. Percentage of DENV‑positive blood cells. B and T cells (CD3^+^CD19^+^) were gated 4 or 5 days after DENV infection. DENV-positive cells were gated compared to the negative control (NC), and shown as a scatter plot. A representative result from three independent experiments is shown. Unpaired two-tailed *t*-test. * *p* < 0.05, ** *p* < 0.01, and *** *p* < 0.001 indicate significant differences. Squares, triangles, and inverted triangles represent the results from DENV-, anti-cEDIII Ab, and anti-EDII-cEDIII Ab-treated groups, respectively.

We next analyzed how inhibition of initial infection by anti-EDII-cEDIII Ab affected virus spread by assaying DENV titers in blood, blood monocytes, and blood lymphocytes (Figure 6). The virus titers in whole blood were non-significantly decreased by both Abs compared to the control, but there was no difference in the serum titers between the control and Ab-treated groups (data not shown). We anticipated that DENV infection would be different in blood cells, because DENV infection was decreased in whole blood but not serum. Because DENV mainly infects monocytes and dendritic cells (DCs), we evaluated the levels of DENV infection in blood cells. DENV infection of monocytes 2 or 3 days after infection was significantly inhibited by anti-EDII-cEDIII Ab compared to the control (Supplementary Figure S2). The reduction in the level of DENV1-infected monocytes was significantly greater in the anti-EDII-cEDIII Ab group than in the anti-cEDIII Ab group. However, DENV infection was not detected in DCs (data not shown).

We detected DENV infection in B and T lymphocytes at 4–5 days after infection, with all four serotypes (Figure 6). At 4 days after infection, anti-EDII-cEDIII Ab significantly reduced the levels of B and T lymphocytes infected with the four serotypes of DENV compared to the control. In addition, the degree of inhibition of DENV infection (all serotypes) of B and T lymphocytes was significantly lower in the anti-EDII-cEDIII Ab group than in the anti-cEDIII Ab group. Therefore, anti-EDII-cEDIII Ab inhibited DENV infection of blood monocytes and B and T lymphocytes.

### 3.7. Anti-EDII-cEDIII Ab Inhibited Secondary Infection of the Liver and Small Intestine

**Figure 7:**
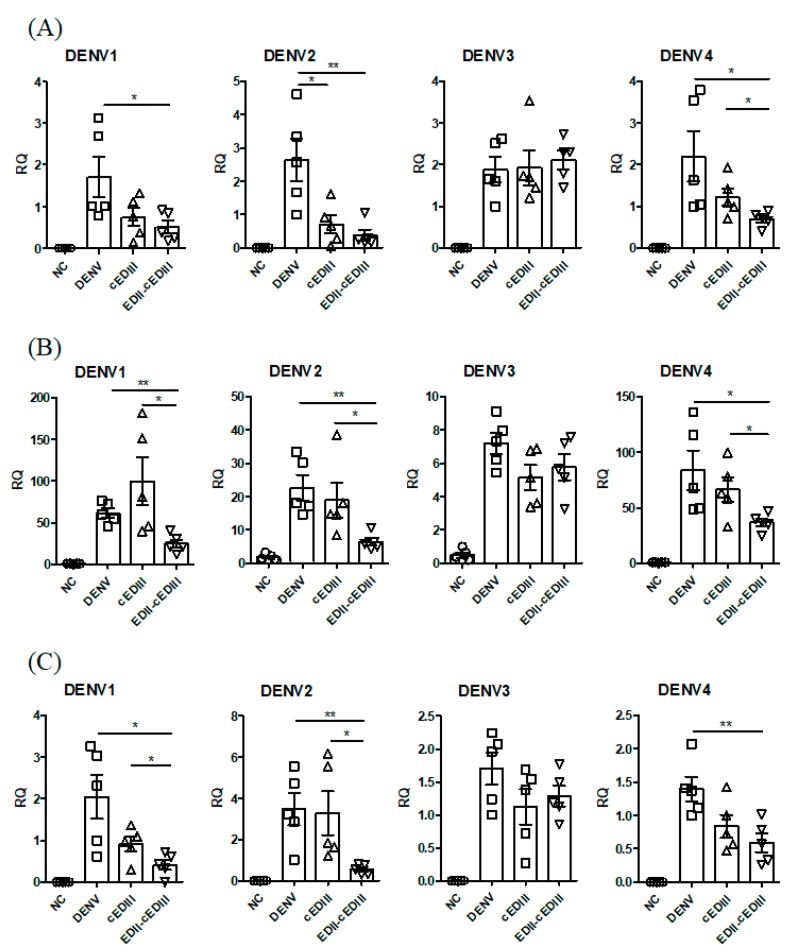
DENV infection at secondary sites is inhibited by purified Abs. Liver tissues collected 5 days after DENV infection with or without the indicated Ab were subjected to RT-qPCR analysis of the levels of (**A**) viral RNA and (**B**) interferon (IFN)-γ. (**C**) Virus in the small intestine was quantified by RT-qPCR at day 5 post-infection. A representative result from two or three independent experiments is shown. Unpaired two-tailed *t*-test. * *p* < 0.05 and ** *p* < 0.01 indicate significant differences. Squares, triangles, and inverted triangles represent the results from DENV-, anti-cEDIII Ab, and anti-EDII-cEDIII Ab-treated groups, respectively.

Given the above results, we postulated that Ab could inhibit the migration of DENV to secondary infection sites. Because DENV was detected in the intestine and liver 3–4 days after infection, we assayed the levels of DENV infection in the liver and small intestine after 4 days [35,36]. Anti-EDII-cEDIII and anti-cEDIII Abs decreased the levels of virus in the livers of mice infected with DENV1, 2, or 4 (Figure 7A). Importantly, anti-EDII-cEDIII Ab, but not anti-cEDIII Ab, significantly reduced DENV1 infection in liver. In addition, anti-EDII-cEDIII Ab significantly decreased DENV4 infection of the liver compared to anti-cEDIII Ab. Anti-EDII-cEDIII Ab also significantly reduced IFN-γ expression in the liver compared to the control, with the exception of DENV3-infected mice (Figure 7B). Moreover, anti-EDII-cEDIII Ab-mediated reduction of IFN-γ expression in mice infected with DENV1, 2, or 4 was significantly greater than that caused by anti-cEDIII Ab. Similarly, anti-EDII-cEDIII Ab significantly reduced the expression levels of DENV genes in the small intestine of mice infected with DENV1, 2, or 4 compared to the control (Figure 7C). In addition, the magnitude of the reduction in expression levels of DENV genes in the small intestines of DENV1- and 2-infected mice caused by anti-EDII-cEDIII Ab was significantly lower than that by anti-cEDIII Ab. Therefore, anti-EDII-cEDIII Ab reduced DENV infection at secondary sites in DENV1, 2, and 4-infected mice, and inhibition by anti-EDII-cEDIII Ab was greater than that by anti-cEDIII Ab.

## 4. Discussion

Dengue is a public health concern globally, and the area suitable for dengue infection and the population at risk of dengue are expected to increase due to environmental changes that promote the replication of host mosquitoes [37]. Following a bite from an infected mosquito, DENV first infects monocytes and DCs expressing mannose receptor or DC-SIGN. The infected cells migrate through lymphatic vessels to LNs, which is considered the initial stage of DENV infection [38,39]. Consequently, monocytes and DCs are important for DENV infection and migration to LNs. Migration of DENV to LNs and the spleen after intraperitoneal infection was also observed in AG129 mice [36]. Anti-EDII-cEDIII Ab decreased DENV infection of LNs and spleen to a greater extent than anti-cEDIII Ab (Figure 5). The reduction in tissue migration of DENV-infected cells was due to inhibition of DENV binding to DC-SIGN receptors by Ab, which interfered with DENV binding to monocytes and DCs. Systemic infection progresses after DENV propagation in initially infected tissues and DENV typically spreads to secondary sites via the bloodstream [38,39]. However, serum virus levels did not differ between mice treated with anti-EDII-cEDIII and anti-cEDIII Abs; monocytes as well as B and T lymphocytes were infected (Figure 6). We believe that these cells are important in systemic DENV infection, because DENV was detected over time after infection. B and T cells can be infected by DENV, and DENV infection activates T cells [40,41]. T cells are permissive to DENV infection and replication, and activated CD4^+^ T cells secrete granzyme A and exert pathogenic effects. In addition, DENV-infected T cells do not undergo apoptosis, which promotes virus replication and maturation [41]. Therefore, inhibition of DENV infection of monocytes and B and T lymphocytes by anti-EDII-cEDIII Ab suggests its potential against DENV infection. Although the anti-EDII-cEDIII Ab inhibited initial DENV infection, DENV3 infection of secondary tissues (Figure 7) and lethality (Figure 4) were not affected compared with the anti-cEDIII Ab. We speculated that this failure to defend against DENV3 was because DENV3 induced the strongest ADE in vitro (Figure 3). We assume that it is difficult to completely avoid ADE in Ab-based experiments, but EDII-cEDIII may assist in the development of an improved recombinant Ag against DENV infection, because the ADE of DENV infection during the initial stage of virus infection was effectively inhibited by anti-EDII-cEDIII Ab compared with anti-cEDIII Ab.

Cytokines produced by local immune cells regulate viral replication and further recruit immune cells to the site of infection. DENV-infected cells migrate to LNs where DENV replicates further and activates B and T cells leading to differentiation of these cells into effector cells. The cytokines produced by these immune cells activate endothelial cells resulting in perturbation of vascular integrity. These cytokines may affect other cell types, including hepatocytes, leading to injury [42]. We also observed changes in the expression of various cytokines during viral infection. Reduced levels of TNF and IL-6 by anti-EDII-cEDIII Ab treatment were observed in the liver during infection of various serotypes of DENV, excluding DENV3 (Supplementary Figure S3). Interestingly, IFN-γ was detected at high levels in the liver during DENV infection, and the difference in the IFN-γ level between anti-EDII-cEDIII and anti-cEDIII Ab treatments was greatest in the liver among the tissues tested, with anti-EDII-cEDIII Ab treatment resulting in significantly lower levels of IFN-γ compared with anti-cEDIII Ab treatment (Figure 7). Since intrahepatic infiltration of CD8^+^ T cells during DENV infection causes liver cell death, this reduction in the IFN-γ level in the liver by anti-EDII-cEDIII Ab is expected to reduce lymphocyte recruitment [43]. Eventually, this reduction may play a role in mitigation of the symptoms caused by DENV infection.

A candidate vaccine effective against DENV is needed. The neutralizing epitopes of DENV have been analyzed by cryogenic electron microscopy. An anti-E protein dimer epitope (EDE) Ab, which recognizes the quaternary structure between E protein dimers, has been found to have a strong neutralizing effect against four serotypes of DENV, to reduce ADE of DENV infection compared to an anti-FLE Ab [34,44]. Recently, an E Ag was engineered to stabilize EDE and limit the exposure of FLE to avoid poor neutralizing activity and the strong infection-enhancing potential of FLE [45]. In this study, we elaborated on a new subunit vaccine candidate by adding EDII (50–130) to cEDIII and assessed its efficacy against DENV infection. The anti-EDII-cEDIII Ab exhibited enhanced binding to DENV4 and neutralization of DENV3 and 4 in Vero E6 cells (Figure 1). It also neutralized all serotypes of DENV in U937, and the effect was superior to that of the anti-cEDIII Ab (Figure 2). We could not rule out the possible exposure of FLE in our EDII-cEDIII Ag, but the anti-EDII-cEDIII Ab showed lower infection enhancement than the anti-cEDIII Ab (Figure 3). Therefore, we hypothesize that EDII promotes the production of DENV-neutralizing Abs. Although EDII-cEDIII did not provide complete protection against all serotypes of DENV, it was more effective than cEDIII. The anti-EDII-cEDIII Ab showed stronger neutralizing activity in U937-DC-SIGN cells than the anti-cEDIII Ab. In addition, initial DENV infection and secondary DENV infection were inhibited more strongly by anti-EDII-cEDIII Ab than by anti‑cEDIII Ab. Collectively, our approach will facilitate the development of Ags for inclusion in a DENV subunit vaccine.

## 5. Conclusions

We developed a candidate vaccine Ag, EDII-cEDIII, which contains partial EDII including the fusion loop and BC loop epitopes together with cEDIII of all four serotypes of DENV. Anti-EDII-cEDIII Ab showed stronger neutralizing activity and lower Ab-dependent peak enhancement of DENV infection compared with anti-cEDIII Ab. Following injection of Ab-treated DENV into AG129 mice, anti-EDII-cEDIII Ab ameliorated DENV infection in tissues with primary and secondary infection more effectively than anti-cEDIII Ab. In addition, anti-EDII-cEDIII Ab protected against DENV1, 2, and 4 challenge. We conclude that EDII-cEDIII is capable of inducing neutralizing and protective Abs and thus shows promise as a candidate subunit vaccine for DENV infection.

## Supplementary Materials

The following are available online at https://www.mdpi.com/2076-393X/8/1/88/s1, Figure S1: ADE by Ab treatment in U937 cells, Figure S2: DENV infection in monocytes, Figure S3: the levels of TNF and IL-6 in liver.
