# Supplementary material for: Addition of Partial Envelope Domain II into Envelope Domain III of Dengue Virus Antigen Potentiates the Induction of Virus-Neutralizing Antibodies and Induces Protective Immunity
Source: Vaccines (Basel). 2020 Feb 15;8(1):88. doi: 10.3390/vaccines8010088 (PMC7157711; doi:10.3390/vaccines8010088)
Supplement: Supplementary file 1 [file vaccines-08-00088-s001.pdf]

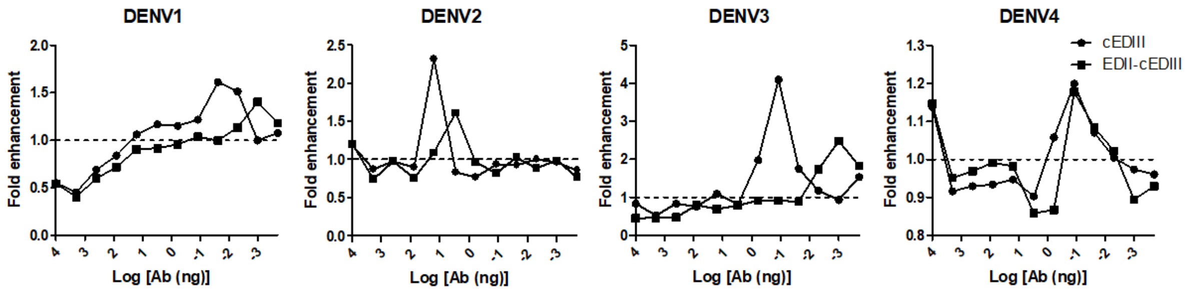

Supplementary Figure S1. Antibody-dependent enhancement (ADE) of DENV infection by Ab treatment in U937 cells. Fold enhancement was determined by comparing the value obtained by Ab treatment with that obtained without Ab treatment. The dotted line represents the fold change relative to DENV infection without Ab treatment.

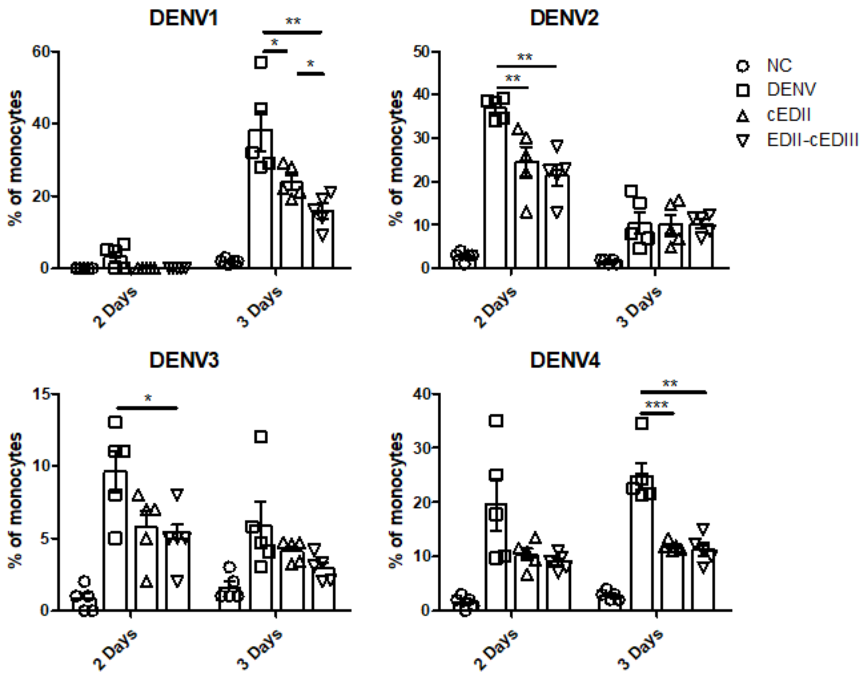

Supplementary Figure S2. Percentage of DENV-positive blood cells. Blood cells were collected 2 and 3 days after infection by DENV and monocytes ($CD3^-CD19^-Ly6G^-Ly6C^+CD11b^+$) were gated by flow cytometry.

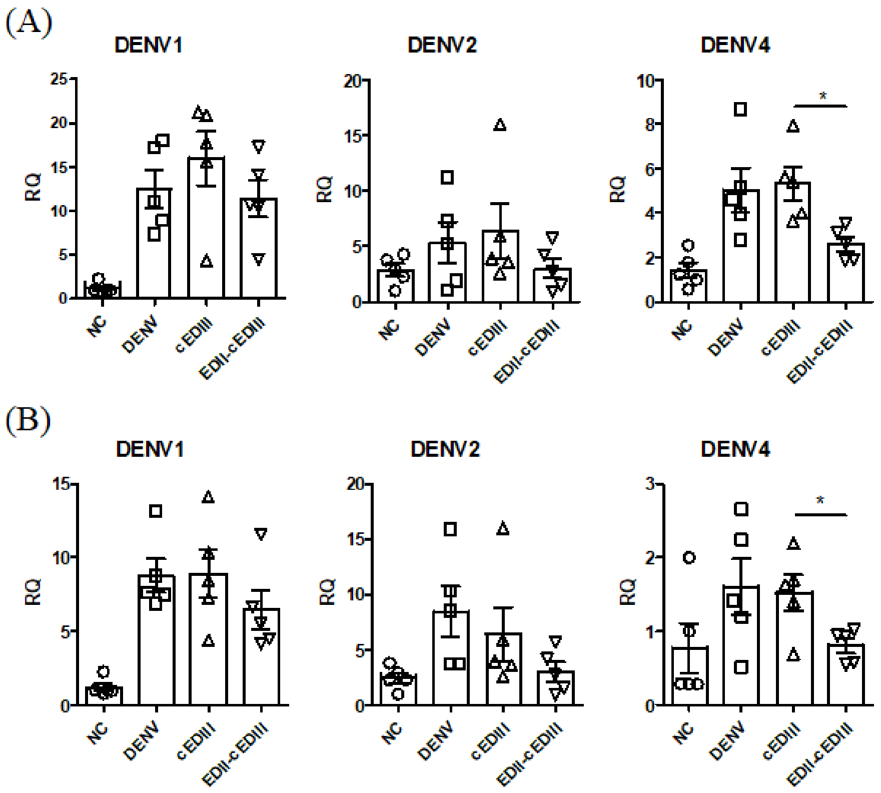

Supplementary Figure S3. DENV infection in secondary tissues is inhibited by purified Abs. Liver tissues collected 5 days after DENV infection with or without the indicated Ab were subjected to qRT-PCR analysis to determine the levels of (A) TNF and (B) IL-6.
